# Pro- and Anti-Inflammatory Cytokines Release in Mice Injected with *Crotalus durissus terrificus* Venom

**DOI:** 10.1155/2008/874962

**Published:** 2008-07-01

**Authors:** A. Hernández Cruz, S. Garcia-Jimenez, R. Zucatelli Mendonça, V. L. Petricevich

**Affiliations:** ^1^Laboratorio de Toxicologia, Facultad de Medicina, Universidad Autónoma del Estado de Morelos (UAEM), Avenida Universidad 1001, Cuernavaca, Morelos 62210, Mexico; ^2^Facultad de Farmacia, Universidad Autónoma del Estado de Morelos (UAEM), Avenida Universidad 1001, Cuernavaca, Morelos 62210, Mexico; ^3^Laboratorio de Imunologia Viral, Instituto Butantan, Anevida Vital Brasil 1500, Butantã 05503000 São Paulo, SP, Brazil

## Abstract

The effects of *Crotalus durissus terrificus* venom (Cdt) were analyzed with respect to the susceptibility and the inflammatory mediators in an experimental model of severe envenomation. BALB/c female mice injected intraperitoneally presented sensibility to Cdt, with changes in specific signs, blood biochemical and inflammatory mediators. The venom induced reduction of glucose and urea levels and an increment of creatinine levels in serum from mice. Significant differences were observed in the time-course of mediator levels in sera from mice injected with Cdt. The maximum levels of IL-6, NO, IL-5, TNF, IL-4 and IL-10 were observed 15 min, 30 min, 1, 2 and 4 hours post-injection, respectively. No difference was observed for levels of IFN-*γ*. Taken together, these data indicate that the envenomation by Cdt is regulated both pro- and anti-inflammatory cytokine responses at time-dependent manner. In serum from mice injected with Cdt at the two first hours revealed of pro-inflammatory dominance. However, with an increment of time an increase of anti-inflammatory cytokines was observed and the balance toward to anti-inflammatory dominance. In conclusion, the observation that Cdt affects the production of pro- and anti-inflammatory cytokines provides further evidence for the role played by Cdt in modulating pro/anti-inflammatory cytokine balance.

## 1. INTRODUCTION

Snake bites represent a serious public health problem in developing countries due to their high incidence, severity, and sequelae [1]. In Brazil, fatal cases of bites involving *Crotalus durissus terrificus* (Cdt) are high, corresponding to 72% of cases not submitted to specific serum treatment and to 11% of cases submitted to specific treatment [2]. This venom contains a variety of toxic proteins including crotoxin, crotamine, gyroxin, convulsium, and thrombin-like enzyme, and produces serious complications, such as neurotoxicity, respiratory paralysis, hypotension, coagulation disorders, myotoxicity, and acute renal failure [3], with possibly additional heart and liver damage [4–6].

Envenomation is involved by a serious, abnormal condition that occurs when an overwhelming infection leads to low blood pressure and low blood flow. The victims may exhibit serious complications, such as disseminated intravascular coagulation and multiple organ failure, and death. With respect to the multipleorgan failure represents effects in endothelial cell injury, oedema formation, and the sequestration and an excessive systemic host inflammatory response are largely mediated by complex immunologic processes. A potent, complex immunologic cascade ensures a prompt protective response to venom in humans and experimental animals. Although activation of the immune system during envenomation is generally protective, septic shock develops in a number of patients as a consequence excessive or poorly regulated immune response to the injury organism. This imbalanced reaction harms the host through a maladaptive release of endogenously generated mediators. A successful immune response is dependent the activation of an appropriate set of immune effectors function and load may determine the differentiation of precursos T helper (Th0) lymphocytes into Th1 and Th2 cells [7]. These two subsets of Th cells differ in the effectors functions and mainly in the repertoire of cytokines that they secrete in response to antigenic stimulation. Th1 cells promote cell-mediated effectors responses, whereas Th2 cells promote B cell-mediated humoral responses. Cytokines produced by Th1 cells include interferon gamma (IFN-*γ*), interleukin-2 (IL-2), IL-12, and tumour necrosis factor beta (TNF-*β*), and constitute a proinflammatory cytokine profile. Those produced by Th2 cells named as anti-inflammatory cytokine include IL-4, IL-5, IL-6, and IL-10. There are also some cytokines such IL-13 and TNF-*α* which are common to both subsets [8].

Many mechanisms are involved in the pathogenesis of envenomation, including release of cytokines. The cytokines are divided into two important groups: the proinflammatory such as IL-1 [7], IL-6 [8, 9], and TNF-*α* [10, 11], and the anti-inflammatory such as IL-10, and have a negative impact on resistance to infection and in septic mice, reduction of IL-10 levels improves survival [12–14].

Cytokine production in envenomation has been widely studied, and it seems that both pro- and anti-inflammatory cytokines are overproduced in sepsis syndrome. However, their clinical significance and prognostic value have not been elucidated [15–19], it seems that a complex network of interactions between different cytokines and possibly other components of the immune response takes place during severe infections. These are accumulating data suggesting that an equilibrium between the pro- and anti-inflammatory responses is important for the final outcome of victims with severe envenomation [18, 19].

The another inflammatory mediator is nitric oxide (NO) that is an important free radical serving as a second messenger in processes including neurotransmission, maintenance of vasodilator tone, and arterial pressure and it has been suggested that cytokine-mediated circulatory shock is caused by activation of the inducible isorform (type II) of NOS [20]. In biological systems, nitric oxide decomposes to nitrite and nitrate, and the cytokine-mediated increases in concentrations of nitrite/nitrate. Modifications in the concentrations of nitrite and nitrate production have been associated with several conditions: severe envenomation [19], septic shock, hypertension, and atheriosclerosis [21].

The purposes of the present study are: (a) to evaluate the susceptibility to the toxic effects of Cdt, (b) to determine the glucose, creatinine, and urea levels following injection with Cdt, (c) to investigate the changes in serum levels of proinflammatory cytokines and anti-inflammatory cytokines in an experimental model of severe envenomation induced in mice by Cdt, and (d) to determine the ratios of pro-/anti-inflammatory cytokines in sera from mice injected with Cdt.

## 2. MATERIALS AND METHODS

### 2.1. Chemicals, reagents, and buffers

Actinomycin D, orthophenyldiamine (OPD), sodium nitrate, N-N-(1-naphthyl)ethylene-diamine dihydrochloride), sulfanilamida, RPMI-1640 medium, and fetal calf serum (FCS) were purchased from Sigma (St. Louis, Mo, USA), murine anti-IL-6 (clones MP5-20F3 and MP5-32C11), recombinant IL-6, murine anti-IFN-*γ* (clones XGM1.2 and R4-6A2), recombinant IFN-*γ*, murine anti-IL-10 (clones JES5-16E3 and SXC-1), recombinant IL-10, murine anti-IL-4 (clones 11B11 and BVD6-24G2), recombinant IL-4, murine anti-IL-5 (clones TRFK5 and TRFK4), and recombinant IL-5 were purchased from BD Biosciences Pharmingen (San Jose, Calif, USA), and recombinant TNF was purchased from Boehring Mannheim (Mannheim, Germany).

### 2.2. Venom

Lyophilized venom of *Crotalus durissus terrificus* (Cdt) was obtained from the Laboratory of Herpetology, Instituto Butantan, São Paulo, Brazil, and stored at −20°C. The venom was dissolved in sterile physiological saline [0.85% (w/v) NaCl solution], immediately before use.

### 2.3. Animals

Female BALB/c mice with different body weights were obtained from an established colony maintained by the Bioterio of Instituto de Biotecnología (UNAM, Mex., USA). The animals were maintained and used under strict ethical conditions according to international recommendations for animal welfare set by Committee Members, (International Society on Toxicology, 1992) [22]. Groups of mice were injected intraperitoneally (i.p.) via with different amounts of Cdt and after different intervals of time the blood was collected from the retroorbital plexus. For the assays to determine the kinetics of cytokines since mortality was of a fraction of the injected animals, the number of mice per experimental group ranged between 5 and 15 to obtain blood samples from at least five mice for each time interval. Mice were bled at 0, 1/4, 1/2, 1, 2, 4, 8 and 24 hours, and sera were separated and stored at −20°C until use.

### 2.4. Lethality

Probit method was used to calculate the lethal dose fifty (LD_50_) of Cdt. Four groups of female BALB/c mice with different body weights 10–12 g; 13–15 g; 16–20 g, and 21–25 g were injected intraperitoneally (i.p.) with increasing doses of venom and the number of mice that died were counted after 24 hours. The number of mice used at each dose lethal was ten.

### 2.5. Measurement of rectal temperature

 Groups of female BALB/c mice with 13–15 g were used to study the effects of Cdt on body temperature in an experimental room for animal behavior, which was maintained at 23–25°C. Each mouse was placed individually in cage (19 × 12 × 11 (depth)), then removed every 15 minutes, held loosely in a small cloth bag, and the core body temperature was measured using a digital thermometer. After each measurement, the mouse was returned to its cage. Mice whose rectal temperature before Cdt administration was below 37°C were not used for experiments. Cdt was administered after the temperature became stable.

### 2.6. Blood biochemical

Groups of female BALB/c mice with 13–15 g were injected i.p. with 0.5, or 1, and/or 2 LD_50_ of Cdt dissolved in 0.1 mL of saline solution. Control mice received 0.1 mL of saline solution. Two hours after injection with Cdt, animals were bled. The blood samples were allowed to stand until they formed a clot and the sera were used in biochemical analysis. The glucose, urea, and creatinine levels present in sera from control mice or injected with Cdt were measured using specific kits (SPINREACT diagnostic, Sant Esteve de Bas, Spain), respectively, according to the manufacturer's protocol.

## 3. MEDIATORS PRODUCTION

### 3.1. Nitrite assay

The nitrite levels in sera from mice were determined as previously described by Schmidt et al., 1989 [23]. Briefly, 40 *μ*L of each mice sera sample were transferred to 96-well plates and mixed with 40 *μ*L of the reduction solution (NADPH 1.25 ng/mL; FAD 10.4 ng/mL; KH_2_PO_4_ 0.125 M) containing 0.5 U of NO^−^_2_ reductase for 2 hours at 37°C. After this time, 80 *μ*L of Griess reagent (1 part 0.1% N-1-naphthyl-ethylene-diamine dihydrochloride in water and 1 part 1% sulfanilamida in 3% concentrated H_3_PO_4_) were added to each well. The mixture was incubated for 5 minutes at room temperature and read at 540 nm in a microplate reader. Concentrations were determined compared with a standard curve of sodium nitrite. The detection limit of the assay was 1 *μ*M nitrite.

### 3.2. Cytokines

The levels of cytokines IL-4, IL-5, IL-6, IL-10, and IFN-*γ* in the serum from BALB/c mice were assayed by a two-site sandwich enzyme-like immunosorbent assay (ELISA) [24]. In brief, ELISA plates were coated with 100 *μ*L (1 *μ*g/mL) of the monoclonal antibodies anti-IL-4, anti-IL-5, anti-IL-6, anti IL-10, or anti-IFN-*γ* in 0.1 M sodium carbonate buffer (pH 8.2) and incubated for 6 hours at room temperature. The wells were then washed with 0.1% phosphate-buffered saline (PBS/Tween-20) and blocked with 100 *μ*L of 10% fetal calf serum (FCS) in PBS for 2 hours at room temperature. After washing, duplicate sera samples of 50 *μ*L were added to each well. After 18 hours of incubation at 4°C, the wells were washed and incubated with 100 *μ*L (2 *μ*g/mL) of the biotinylated monoclonal antibodies anti-IL-4, anti-IL-5, anti-IL-6, anti-IL-10, or anti-IFN-*γ* as second antibodies for 45 minutes at room temperature. After a final wash, the reaction was developed by the addition of orthophenyldiamine (OPD) to each well. Optical densities were measured at 405 nm in a microplate reader. The cytokine content of each sample was read from a standard curve established with the appropriate recombinant cytokines (expressed in picograms per millilitre). The minimum levels of each cytokine detectable in the conditions of the assays were 10 pg/mL for IL-4, IL-5, IL-6, and IL-10 and 300 pg/mL for IFN-*γ*.

To measure the cytotoxicity of TNF present in the serum from BALB/c mice, a standard assay with L-929 cells, a fibroblast continuous cell line was used as described previously by Ruff and Gifford (1988) [25]. The percentage cytotoxicity was calculated as follows: (A_control_−A_sample_/A_control_)×100. Titres were calculated as the reciprocal of the dilution of the sample in which 50% of the cells in the monolayer were lysed. TNF activity is expressed as pg/mL, estimated from the ratio of a 50% cytotoxic dose of the test to that of the standard mouse recombinant TNF.

### 3.3. Statistical analysis

Data are expressed as the mean ± standard deviation. Statistical analyses were performed by Student *t*-test and the level of significance was set at *P* < .05.

## 4. RESULTS

### 4.1. Determination LD_50_ and symptoms

**Figure 1:**
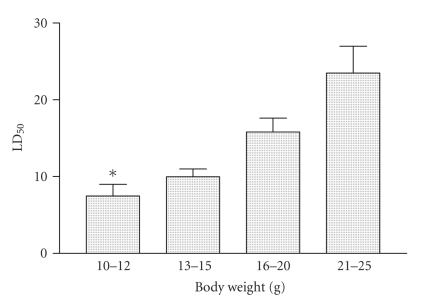
*Effect of Cdt according to body weight*. Groups of 10 BALB/c female mice with different body weight were i.p. injected with different amounts of Cdt. Deaths occurring during 24 hours were recorded and the LD_50_ value was calculated. Statistical difference between the groups were marked with asterisk (*P* < .01).

To verify whether the venom present an effect on the body weight and also to determine the LD_50_, groups of female BALB/c mice with different body weight were injected intraperitoneally with distinct doses of Cdt. The LD_50_ value was calculated by probit analysis at 95% confidence. These animals were distributed in four groups, with different body weights. As shown in Figure 1, BALB/c female mice presented different susceptibility to Cdt, (10–12 g, LD_50_ = 7.5 *μ*g), (13–15 g, LD_50_ = 10 *μ*g), (16–20 g, LD_50_ = 15.8 *μ*g), and (21–25 g, LD_50_ = 23.5 *μ*g). The highest susceptibility was observed for female groups with 10–12 g of body weights. As body weight increased, it was possible to observe a decrease in susceptibility (Figure 1). When mice received an intraperitoneal injection of 1 LD_50_ of Cdt, the time course of mortality did not differ between the groups studied. In all groups, the majority of deaths occurred within first 6 hours. No deaths were observed in mice injected with saline solution (results not shown). Thus, in subsequent experiments mice weighting 13–15 g were used.

**Figure 2:**
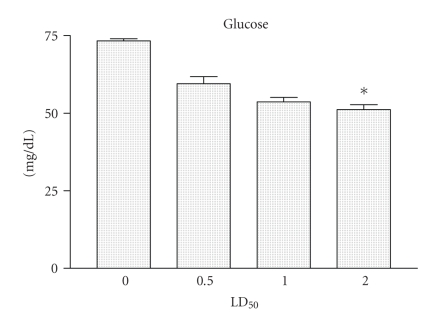
*Glucose determination*. Groups of female mice from the BALB/c strain, 13–15 g of body weight, were injected i.p. with 0.5, or 1, and/or 2 LD_50_ of Cdt. After 2 hours the mice were bled and the levels of glucose present in sera from mice injected were determined. Each vertical bar represents the mean plus or minus the standard deviation value of samples from the results obtained with two experiments carried out with two independent groups of five mice each. Statistical differences between the injections were marked with asterisk (*P* < .01).

Death was usually preceded by certain signals or symptoms such as hypothermia. Groups of BALB/c female mice of 13–15 g of body weight were injected i.p. with 0.5, or 1, and/or 2 LD_50_ of Cdt, and at different intervals of time specific signs were observed (data not shown). To determine the glucose, urea, and creatinine levels, groups of BALB/c female mice with 13–15 g of body weight were injected i.p. with 0.5, or 1, and/or 2 LD_50_ of Cdt for 2 hours. As shown in Figure 2 the LD_50_ increased, it was possible to observe a decrease in glucose levels. The glucose levels were significantly lower for animal groups that received Cdt when compared with those obtained from control groups of animals (*P* < .01).

**Figure 3:**
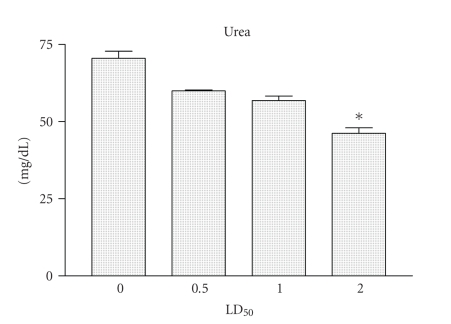
*Urea determination*. Groups of female mice from the BALB/c strain, 13–15 g of body weight, were injected i.p. with 0.5, or 1, and/or 2 LD_50_ of Cdt. After 2 hours the mice were bled and the urea levels present in sera were determined. Each vertical bar represents the mean plus or minus the standard deviation value of samples from the results obtained with two experiments carried out with two independent groups of five mice each. Statistical differences between the injections were marked with asterisk (*P* < .01).

Figure 3 shows that all mice that received different amounts of Cdt, the levels of urea in sera were significantly lower (*P* < .01) when compared with those obtained for control group.

**Figure 4:**
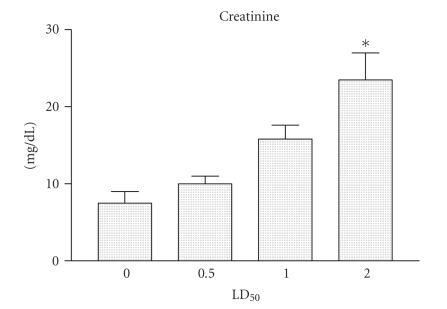
*Creatinine determination*. Groups of female mice from the BALB/c strain, 13–15 g of body weight, were injected i.p. with 0.5, or, 1 and/or 2 LD_50_ of Cdt. After 2 hours the mice were bled and the creatinine levels present in sera were determined. Each vertical bar represents the mean plus or minus the standard deviation value of samples from the results obtained with two experiments carried out with two independent groups of five mice each. Statistical differences between the injections were marked with asterisk (*P* < .01).

The levels of creatinine in sera from groups of mice injected with Cdt are shown in Figure 4. The levels of creatinine were increasing in a concentration-dependent manner. The maximum levels of creatinine were observed in sera from groups of mice injected with 2 LD_50_ (Figure 4).

### 4.2. Comparative in vivo mediators release upon Cdt venom injection

**Figure 5:**
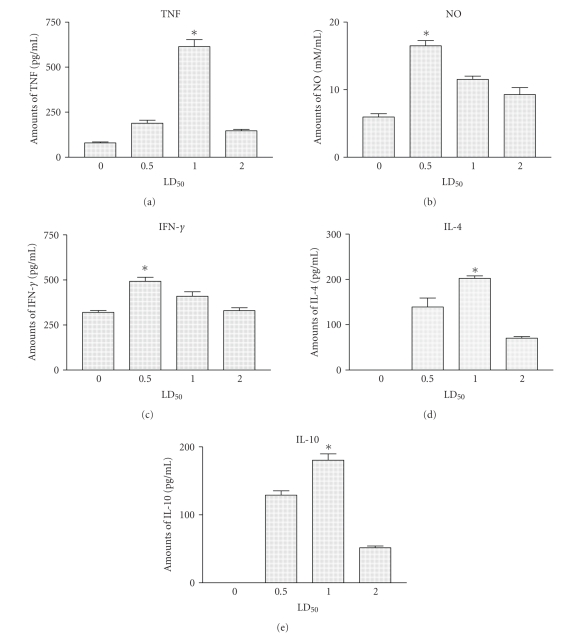
*Mediators secretion*. Groups of BALB/c female mice with 13–15 g were i.p. injected with 0.5, or, 1 and/or 2 LD_50_ of Cdt, after 2 hours, the animals were bled and the levels of cytokines and NO present in the serum were determined as described in materials and methods (see Section 2). Each point represents the mean value of the results obtained from two independent experiments conducted with five to fifteen animals each. Statistical differences between the injections were marked with asterisk (*P* < 0.001).

In order to compare the mediators release such as cytokine secretion and nitric oxide production, sera from BALB/c female mice with 13–15 g of body weight were injected i.p. with 0.5, or, 1 and/or 2LD_50_ of Cdt and bled after 2 hours. At this time, the levels of IL-5 and IL-6 were undetectable in serum from mice injected with different LD_50_. As shown in Figure 5, the levels of TNF, NO, IL-4, and IL-10 were significantly higher (*P* < .001) in sera from mice injected for 2 hours with different amounts of Cdt when compared with those obtained in sera from control group. Interestingly the results obtained also shown that the levels of these mediators were consistently and significantly lower (*P* < .001) in sera from mice injected with 2 LD_50_ when compared to those obtained in sera from groups of mice that received 0.5 and/or 1 LD_50_ (Figure 5). In contrast, no significant difference was observed in the levels of IFN-*γ* present in sera from mice injected with different amounts of Cdt (Figure 5).

### 4.3. Kinetic of mediators release upon Cdt venom injection

**Figure 6:**
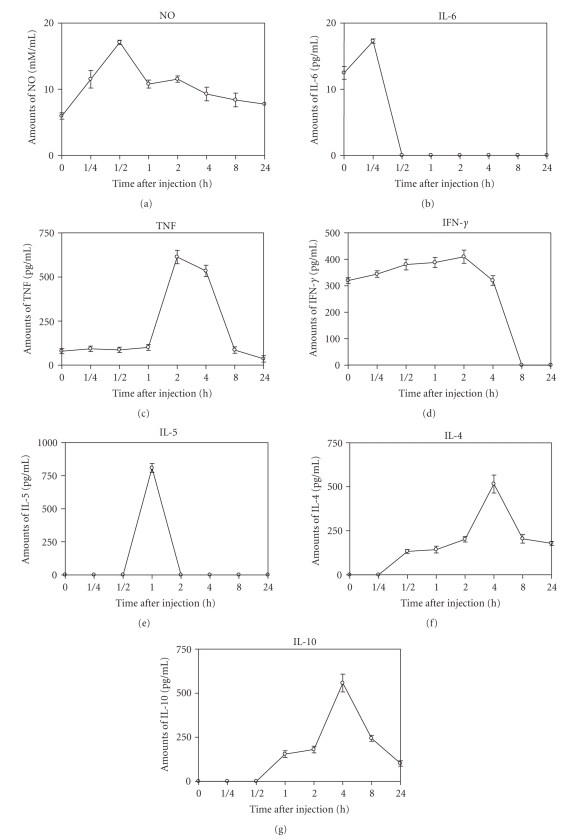
*Kinetics of mediator secretion*. Groups of female mice from BALB/c strain, 13–15 g of body weight, were i.p. injected with 1 LD_50_ of Cdt. After different times the animals were bled and the levels of cytokines present in the serum were determined as described in materials and methods (see Section 2). Each point represents the mean value of the results obtained from two independent experiments conducted with five to fifteen animals each. Statistical differences between the injections were (*P* < .001).

To determine the kinetic of cytokine secretion and NO production, groups of BALB/c female mice with 13–15 g of body weight were injected i.p. with 1 LD_50_ of Cdt and bled after different time intervals. The highest levels of NO^−^_2_ after Cdt injection were observed at 30 minutes postinjection, decaying thereafter (Figure 6). Cdt induced a discrete increment of IL-6 levels at 15 minutes postinjection (Figure 6). The TNF and IFN-*γ* levels increased gradually, reaching their highest at 2 hours postinjection, decaying thereafter (Figure 6). The highest levels of IL-5 were observed at 1 hour postinjection (Figure 6). Cdt was also capable to induce an increase in the serum levels of IL-4 and IL-10 with the highest values occurring 4 hours postinjection, decaying thereafter (Figure 6).

### 4.4. Effect of Cdt on pro-/anti-inflammatory cytokines balance

**Figure 7:**
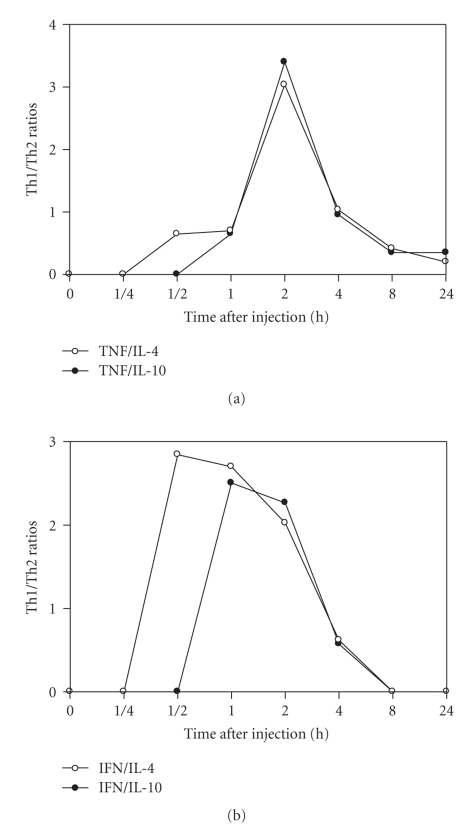
*Pro-/anti-inflammatory cytokine balance*. The serum levels of cytokines were determined as described in materials and methods (see Section 2). The ratios of pro-/anti-inflammatory; IFN-*γ*/IL-4, IFN-*γ*/IL-10, TNF-*α*/IL-4, and TNF-*α*/IL-10 represent the values of samples from two experiments in different groups of five to fifteen mice.

Cytokines were determined as above described and to investigate the changes in serum levels of proinflammatory cytokines (IFN-*γ* and TNF-*α*) and anti-inflammatory cytokines (IL-4 and IL-10) and the ratios of pro-/anti-inflammatory (TNF-*α*/IL-4, TNF-*α*/IL-10, IFN-*γ*/IL-4, and IFN-*γ*/IL-10) were calculated. As shown in Figure 7, the TNF-*α*/IL-4 and TNF-*α*/IL-10 ratios increased gradually, reaching its highest at 2 hours, decaying thereafter. The highest ratios IFN-*γ*/IL-10 and IFN-*γ*/IL-4 were observed at 15 minutes until 2 hours, respectively (Figure 7). The TNF-*α* and IFN-*γ* reduction were accompanied by increased IL-4 and IL-10 release.

## 5. DISCUSSION

Various factors can contribute to the presence of specific signs and symptoms followed by stings or bites with respect to the venom toxicity variations [26]. However, it has been demonstrated that other factors may also contribute to clinical signs, such as age or size of the victims, the site of the injection, and the vulnerability of the victim to the venom [15, 26, 27].

The present study was designed to simulate accidental envenomation in humans, wherein the route of Cdt administration, the time elapsed between the injection and specific signs, the dose administered, and mediators production were studied. The experimental models studied should involve different susceptibility to the venom toxic effects. This was achieved in the present study, the highest susceptibility was observed for female groups at different body weights. Among the analyzed of female BALB/c within 10–12 g was significantly more susceptible to the Cdt lethal effects than the other groups with different body weights. In the present study, we observed that mice presented respiratory abnormalities following Cdt injection. These observations agree with previous studies that showed that Cdt produces respiratory abnormalities in mice [28].

Various studies carried out show that *Crotalus* venom induces in animals generalized rhabdomyolysis, causing myalgias, by the increment massive rise in serum of myoglobin and creatinine kinase levels accompanied by myoglobinuria [29]. Acute renal failure is the main cause of death among humans observed after de envenomation with Cdt and possible additional heart and liver damage [2–6]. In this study, we observed changes in several blood biochemical parameters in the mice were measured after the Cdt injection. The amounts of serum glucose, urea, and creatinine levels measurements are described in detail in Figures 2, 3, and 4. These results agree with previous studies that showed that clinical and laboratory alterations in animals immunized with snake venoms [30]. Cdt envenomation also presents an elevation of catecholamines, angiotensin II, glucagons, and cortisol accompanied by changes in insulin secretion [31]. In the *Crotalus* envenomation the insulin and glucose metabolism alterations could be responsible for the pathogenesis of variety of clinical manifestations. The present study showed that in the blood of groups of mice injected with Cdt, the levels of glucose were decreased. Urea is formed in the liver and circulates in the blood in the form of urea nitrogen. In healthy humans most urea nitrogen is filtered out by the kidneys and leaves the body in urine. If kidneys are not functioning properly or if the body is using large amounts of protein, the blood urea nitrogen level rises. If the human has severe liver disease the blood urea nitrogen will decay. In present study, we observed decreased levels of urea in blood from mice injected with Cdt that suggested a liver failure. These results are inline with previous reports showing that human patients who were bitten by Cdt showed hydropic degeneration and mitochondrial injury in the liver [4]. Changes in blood parameters which are typical effects of Cdt were glucose and urea levels decreased whereas creatinine increased. The present study also shows that these alterations in serum were observed only when large amounts of Cdt were injected into the animals.

The envenomation is characterized by a generalized inflammatory state. The normal reaction to envenomation involves a series of complex immunologic cascade that ensures a prompt protective response to venom in humans [32] and experimental animals [15–19, 27]. Although activation of the immune system during envenomation is generally protective, the septic shock develops in a number of patients as a consequence of excessive or poorly regulated immune response to the injury organism [19, 32]. This imbalanced reaction may harm the host through a maladaptitive release of endogenous mediators that include cytokines and nitric oxide.

Cytokines are soluble protein mediators important for the orchestration of inflammatory responses of the human body [33]. The production of proinflammatory and anti-inflammatory cytokines is strictly controlled by complex feedback mechanisms [14, 34, 35]. Cytokines may be divided into proinflammatory and anti-inflammatory. The proinflammatory cytokines such TNF-*α*, IL-1 and IL-8 that include the mobilizing immune system cells to proliferate and produce more cytokines creating an inflammatory cascade, and as anti-inflammatory cytokines such IL-10 which function to dampen or control the inflammatory response. Proinflammatory cytokines are primarily responsible for initiating a potent defence against exogenous pathogens. In contrast, anti-inflammatory cytokines are crucial for down regulating the elevated inflammatory process and maintaining homeostasis for the correct functionality of vital organs [36]. However, excessive production of these mediators may significantly contribute to shock, multiple organ failure, and death [14, 34, 35].

Envenomation is a constellation of clinical signs and symptoms resulting from excessive systemic host inflammatory response that are largely mediated by cytokines, which are released into the systemic circulation. Serum concentrations of specific cytokines TNF-*α*, IL-6 that are frequently elevated in envenomated mice and their concentrations correlate with severity and outcome of envenomation. In addition, proinflammatory cytokines are produced in large quantities in envenomated mice, however, the specific role of these molecules in sepsis remains undefined.

A balanced ratio of pro- and anti-inflammatory cytokines is important for appropriate immune response, excessive inflammation, or hyporesponsiveness which can lead to envenoming complications. To determine the magnitude of the cytokine response caused by Cdt venom injection and to evaluate the balance of pro- and anti-inflammatory cytokines released during the envenomation, we measured levels of cytokines in serum from mice.

TNF-*α* is a proinflammatory cytokine which plays an important role in the immune response to infections and cancer and in the regulation of inflammation [37]. The present study shows that the elevation of serum concentrations of TNF-*α* which occurs 2 hours after of Cdt administration.

IL-6 is produced by a variety of cell types during infection, trauma, and immunological challenge. The functional properties of IL-6 are extremely varied and this is reflected by the terminology originally used to describe the activities of this cytokine. It has been described to have both pro- and anti-inflammatory effects, as well as being involved in a variety of immune response. The results obtained in this study showed that the levels of IL-6 increased until 15 minutes after the 1 LD_50_ Cdt injection, decaying thereafter.

IFN-*γ* is produced by a variety of cell types and probably plays a role in the early stages of host response to venoms. In the present study the serum concentrations of IFN-*γ* were similar for all groups of mice injected with different amounts of Cdt. In groups of mice injected with 1 LD_50_ of Cdt the levels of IFN-*γ* were possibly observed a modest until 4 hours, decaying thereafter.

 IL-10 is a pluripotent immunoregulatory cytokine that has not been previously characterized in T-cell clones from humans and mice. IL-10 is an anti-inflammatory cytokine that potently inhibits the proinflammatory cytokines secretion such as TNF and IL-1 [38] and regulates the differentiation and proliferation of several immune cells [39]. The present study also shows that the levels of IL-10 increased until 4 hours in groups of mice injected with 1 LD_50_ of Cdt.

IL-4 has a wide range of functions and in vivo this cytokine is principally responsible for the production of IgE in mice in response to a variety of stimuli that elicit Ig class switching to the expression of this Ig class [40]. The present study showed that Cdt has the ability to stimulate the IL-4 production that certainly is exerting a modulatory effect of host inflammatory response.

In this study, we observed that the levels of all mediators with exception of IFN-*γ* were consistently and significantly lower (*P* < .001) in sera from mice injected with 2 LD_50_ when compared to those obtained in sera from groups of mice that received 0.5 and/or 1 LD_50_ (Figure 5). These results agree with previous studies that are carried out the crotoxin that is the major neurotoxin present in *Crotalus* venom, demonstrated the activities such as immunosuppresor and immunomodulatory in experimental animals [41, 42].

NO is known to be involved in multiple biologically important reactions [43, 44]. This chemical compound is a gas that easily diffuses from the endothelial cells to the smooth muscle cells on the vascular wall. The present study showed that the Cdt has the ability to stimulate the NO production that certainly is exerting a modulatory effect on the host inflammatory response. The production of NO is one of the main mechanisms involved in endothelium function. When NO is synthesized from arginine, by the NO synthase (NOS) reaction, citrulline and intermediate product of the urea cycle is formed. Thus, the urea cycle is bypassed by the NOS reaction. With respect to the levels of mediator, similar results were obtained for mice groups with different body weight (data not shown).

In this study, we showed that the levels of IFN-*γ* and TNF-*α* were higher in mice injected with 1 LD_50_ than in control group and/or mice group injected with 2 LD_50_. Nevertheless, a direct correlation between IFN-*γ* and TNF-*α* and IL-4 and IL-10 cytokines was observed in mice injected with Cdt indicating a mutual pro-/anti-inflammatory participation. In conclusion, Cdt is regulated by both pro- and anti-inflammatory cytokine responses. In groups of mice injected by short period of time the deviation of the pro-/anti-inflammatory balance toward to pro-inflammatory predominant type. In contrast, with the increasing of injection time the deviation of balance was to anti-inflammatory dominance.
